# Prognostic model for predicting recurrence-free survival in HBV-related hepatocellular carcinoma patients after combined treatment: a multicenter study

**DOI:** 10.3389/fonc.2026.1760126

**Published:** 2026-05-04

**Authors:** Bojun Liu, Xue Yin, Wenying Qiao, Xiaoyan Ding, Caixia Hu

**Affiliations:** 1 Interventional Therapy Center for Oncology, Beijing You’an Hospital, Capital Medical University, Beijing, China; 2 National Center for Infectious Diseases, Beijing Ditan Hospital, Capital Medical University, Beijing, China; 3 Department of Infectious Disease, The Third Xiangya Hospital, Central South University, Changsha, Hunan, China

**Keywords:** hepatitis B virus, hepatocellular carcinoma, least absolute shrinkage and selection operator, random survival forest, recurrence-free survival

## Abstract

**Introduction:**

Hepatocellular carcinoma (HCC) is a lethal malignancy, with hepatitis B virus (HBV) infection as its leading cause in China. Transarterial chemoembolization (TACE) combined with radiofrequency ablation (RFA) treatment was gradually applied in clinic, but the lack of targeted prognostic tools for this cohort remains a critical issue. This multicenter study aimed to develop a prognostic model for recurrence-free survival (RFS) in HBV-related HCC patients after the combined treatment.

**Methods:**

A total of 604 patients from two hospitals were enrolled; 502 (Beijing You’an Hospital) formed the training/internal validation cohort (7:3 split), and 102 (Beijing Ditan Hospital) served as the external validation cohort. Baseline clinical, tumor, and laboratory data were collected. LASSO regression and random survival forest were used for variable screening, followed by multivariate Cox regression to identify independent predictors. The model was visualized as a nomogram, evaluated via Kaplan-Meier survival curves, receiver operating characteristic (ROC) curves, and decision curve analysis (DCA).

**Results:**

Age, tumor number, tumor size, gamma-glutamyl transferase (GGT), and total bilirubin (TBIL) were identified as independent RFS factors. The nomogram developed based on the five factors exhibited good discriminative ability: AUC values for 1-, 3-, 5-year RFS were 0.759, 0.777, 0.783 (training cohort), 0.708, 0.751, 0.714 (internal validation cohort), and 0.701, 0.708, 0.751 (external validation cohort). Kaplan-Meier curves confirmed significant RFS differences between high/low-risk groups (all P<0.001), and DCA demonstrated positive net clinical benefit.

**Conclusion:**

This study successfully developed a well-performed nomogram model to predict 1-, 3-, and 5-year RFS in HBV-related HCC patients following TACE combined with RFA.

## Introduction

Hepatocellular carcinoma (HCC) remains one of the most prevalent and lethal malignancies worldwide, accounting for approximately 75%-85% of primary liver cancer cases, which ranks fourth among cancer-related deaths globally (1, 2). The global burden of liver cancer continues to rise, with an estimated 866,136 new cases and 758,725 deaths reported in 2022 (3). In China, hepatitis B virus (HBV) infection is the main cause of HCC, posing a severe threat to public health (4). The clinical management of HCC is complex and highly dependent on tumor stage, liver functional reserve, and patient status (5). For HCC patients, a range of curative and palliative options exists. Among these, interventional therapies, particularly transarterial chemoembolization (TACE) and radiofrequency ablation (RFA), have become cornerstones of treatment (6–8). In clinic, the combined use of TACE and RFA has emerged as an increasingly common therapeutic approach for HCC (9, 10).

Despite the development in the treatment approaches, the long-term prognosis for HCC patients remains suboptimal, primarily due to the high incidence of tumor recurrence (11). Accurately stratifying patients based on their individual recurrence risk is crucial for identifying high-risk candidates who may benefit from more intensive follow-up protocols. Current prognostic assessment for HCC primarily relies on conventional staging systems such as Barcelona Clinic Liver Cancer (BCLC) (12), which integrate tumor burden, liver function, and general status but lack specificity for HBV-related HCC patients receiving TACE combined with RFA therapy. Additionally, although several prognostic models have been established (13, 14), they also fail to possess targeted applicability for this particular patient cohort.

A critical challenge in prognostic model construction lies in selecting high-value predictive variables from a large pool of clinical and laboratory indicators while avoiding overfitting. Least absolute shrinkage and selection operator (LASSO) regression effectively addresses this by performing variable selection and regularization simultaneously, shrinking irrelevant variables to zero (15). Complementarily, random survival forest (RSF), a non-parametric ensemble method, excels in capturing non-linear relationships and interactions between variables, and demonstrates robust performance in survival analysis with complex data structures (16). The combination of LASSO and RSF thus offers a synergistic approach to enhance the accuracy and stability of variable screening.

This multicenter study aims to identify independent prognostic factors for recurrence-free survival (RFS) in HBV-related HCC patients after TACE combined with RFA treatment based on advanced techniques and develop a practical prognostic model, ultimately providing a clinical decision-making tool to optimize post-treatment surveillance and intervention strategies.

## Materials and methods

### Study patients

This retrospective study enrolled 604 HBV-related HCC patients between January 2015 and January 2022, all receiving a combined treatment of TACE and RFA. Among them, 502 patients were from Beijing You’an Hospital affiliated to Capital Medical University, and 102 patients were from Beijing Ditan Hospital affiliated to Capital Medical University. This study obtained approval (ethical approval number: LL-2023-131-K) from both the ethics committee of Beijing You’an Hospital and the ethics committee of Beijing Ditan Hospital. Due to the retrospective design and the use of anonymized data, the requirement for informed consent was waived.

Patient selection was based on predefined inclusion and exclusion criteria. Eligible subjects were required to have a confirmed diagnosis of HBV-related HCC, have received combined TACE and RFA treatment, and have complete clinical and follow-up data. The diagnosis of HCC was established based on the criteria of the American Association for the Study of Liver Diseases (AASLD), involving typical imaging characteristics on contrast-enhanced computed tomography (CT)/magnetic resonance imaging (MRI), and/or pathological confirmation. HBV-related HCC is defined as HCC that develops in the context of chronic HBV infection, mainly based on positive serum hepatitis B surface antigen (HBsAg). Exclusion criteria encompassed non-primary HCC, non HBV-related HCC, prior anti-tumor therapies, coexistence of other malignancies, and incomplete medical or follow-up records. All treatments were performed by experienced physicians with at least five years of expertise. The therapeutic sequence consisted of initial TACE, aimed at delivering chemotherapeutic and embolic agents directly to tumor-feeding blood vessels, followed by RFA within 1 to 2 weeks.

### Data collection

The baseline data of patients before receiving the combination therapy were collected, mainly including basic personal information, tumor features, and laboratory test results. Basic personal information included age, gender, hypertension status, diabetes status, cirrhosis status, antiviral history, smoking history and family history. Tumor features consisted of Child-Pugh grade, BCLC stage, tumor number, tumor size, and alpha-fetoprotein (AFP) levels. Laboratory parameters covered a wide range, including complete blood count with components such as white blood cells (WBC), neutrophils, lymphocytes, monocytes, red blood cells (RBC), hemoglobin, and platelets; liver function variables including alanine aminotransferase (ALT), aspartate aminotransferase (AST), AST/ALT ratio, total bilirubin (TBIL), direct bilirubin (DBIL), total protein, albumin, globulin, gamma-glutamyl transferase (GGT), alkaline phosphatase (ALP), prealbumin, and bile acid; coagulation profile parameters including prothrombin time (PT), activated partial thromboplastin time (APTT), fibrinogen, and thrombin time (TT); renal and metabolic markers such as uric acid, glucose, and cholesterol; electrolytes including potassium, sodium, and chloride.

### Patients follow up

All patients were advised to complete regular follow-up assessments following treatment initiation. The first follow-up visit was arranged at the outpatient clinic approximately one month post-treatment to evaluate therapeutic response. For the subsequent follow-up schedule, patients were monitored quarterly (every 3 months) during the first year after treatment, and this frequency was adjusted to biannual (every 6 months) thereafter until tumor recurrence was identified or follow-up was discontinued. Tumor recurrence was defined as the detection of new hepatic lesions via contrast-enhanced CT or MRI, with confirmation by pathological examination if necessary. RFS was calculated as the time interval from the date of treatment completion to the date of documented recurrence or the last available follow-up date, and was recorded in months. The final follow-up date for this study was January 1, 2025.

### Statistical analysis

Statistical computations were performed utilizing R software (version 4.3.2), with a P value of less than 0.05 defined as statistically significant. 502 patients recruited from Beijing You’an Hospital constituted the initial study population, who were randomly allocated into the training cohort and internal validation cohort at a proportion of 7:3. For the purpose of identifying pivotal factors associated with RFS in HCC patients after combination treatment, multiple statistical approaches were employed, namely LASSO regression, RSF, and multivariate Cox proportional hazards regression. Subsequently, a nomogram was constructed to predict post-treatment RFS based on the critical prognostic factors identified through the aforementioned analyses. To comprehensively evaluate the discriminative ability and clinical utility of the established nomogram, several statistical assessments were adopted, including Kaplan-Meier survival curves, receiver operating characteristic (ROC) curves, calibration curves, and decision curve analysis (DCA). Furthermore, an external validation cohort consisting of 102 HCC patients from Beijing Ditan Hospital was used to further verify the model’s performance and generalizability. With respect to data presentation and between-group comparison, continuous variables were summarized as mean ± standard deviation, and group differences were analyzed using Student’s t-test. Categorical variables were presented as frequencies and percentages, and the Chi-square test was applied to compare the distribution differences between groups.

## Results

### Baseline characteristics of the patients

This study included 604 HBV-related HCC patients who underwent combined TACE and RFA treatment between January 2015 and January 2022. Among them, 502 patients from Beijing You’an Hospital affiliated with Capital Medical University were randomly assigned to a training cohort and an internal validation cohort in a 7:3 ratio, while 102 patients from Beijing Ditan Hospital served as the external validation cohort. The baseline demographic, clinical, and laboratory characteristics of the patients across the three cohorts are summarized in **Table 1**, with no statistically significant differences observed among the groups (all P > 0.05), supporting their suitability for model development and validation. In terms of demographic features, the mean age of participants in all three cohorts ranged from approximately 55 to 57 years. Males constituted the majority (65.7%-73.5%) in each cohort. The distribution proportions of underlying conditions such as hypertension and diabetes, as well as smoking history, antiviral treatment history, and family history, were similar across the three groups. Regarding disease-related characteristics, patients with cirrhosis accounted for the majority (85.3%-89.4%) in all three cohorts. Most patients were classified as Child-Pugh Grade A (69.6%-79.5%), and over 80% of patients were in BCLC Stage 0 or A.

**Table 1:** Baseline characteristics of training, internal validation, and external validation cohorts.

Characteristics		Training cohort (n=351)	Internal validation cohort (n=151)	External validation cohort (n=102)	P value
Age		56.83 ± 9.23	55.34 ± 10.42	57.08 ± 8.97	0.22
Gender	Male	248 (70.7%)	111 (73.5%)	67 (65.7%)	0.407
	Female	103 (29.3%)	40 (26.5%)	35 (34.3%)	
Hypertension	No	262 (74.6%)	119 (78.8%)	79 (77.5%)	0.571
	Yes	89 (25.4%)	32 (21.2%)	23 (22.5%)	
Diabetes	No	291 (82.9%)	127 (84.1%)	79 (77.5%)	0.355
	Yes	60 (17.1%)	24 (15.9%)	23 (22.5%)	
Antiviral_history	No	150 (42.7%)	58 (38.4%)	44 (43.1%)	0.633
	Yes	201 (57.3%)	93 (61.6%)	58 (56.9%)	
Smoking_history	No	242 (68.9%)	102 (67.5%)	79 (77.5%)	0.191
	Yes	109 (31.1%)	49 (32.5%)	23 (22.5%)	
Family_history	No	179 (51.0%)	89 (58.9%)	55 (53.9%)	0.261
	Yes	172 (49.0%)	62 (41.1%)	47 (46.1%)	
Cirrhosis	No	44 (12.5%)	16 (10.6%)	15 (14.7%)	0.62
	Yes	307 (87.5%)	135 (89.4%)	87 (85.3%)	
ChildPugh	A	279 (79.5%)	117 (77.5%)	71 (69.6%)	0.111
	B	72 (20.5%)	34 (22.5%)	31 (30.4%)	
BCLC	0	116 (33.0%)	53 (35.1%)	27 (26.5%)	0.66
	A	187 (53.3%)	77 (51.0%)	61 (59.8%)	
	B	48 (13.7%)	21 (13.9%)	14 (13.7%)	
Tumor_number	single	252 (71.8%)	105 (69.5%)	76 (74.5%)	0.688
	multiple	99 (28.2%)	46 (30.5%)	26 (25.5%)	
Tumor_size	≤3cm	249 (70.9%)	104 (68.9%)	69 (67.6%)	0.778
	>3cm	102 (29.1%)	47 (31.1%)	33 (32.4%)	
WBC (10^9/L)		5.17 ± 1.96	5.16 ± 2.20	5.08 ± 2.29	0.926
Neutrophil (10^9/L)		3.31 ± 1.64)	3.37 ± 1.86	3.33 ± 2.05	0.94
Lymphocyte (10^9/L)		1.35 ± 0.96	1.21 ± 0.56	1.20 ± 0.55	0.116
Monocyte (10^9/L)		0.41 ± 0.21	0.43 ± 0.24	0.41 ± 0.28	0.758
RBC (10^9/L)		4.20 ± 0.57	4.16 ± 0.64	4.13 ± 0.70	0.595
Platelet (10^9/L)		126.73 ± 61.22	125.90 ± 64.79	125.25 ± 62.76	0.974
Total_protein (g/L)		65.75 ± 6.28	64.72 ± 8.29	64.18 ± 8.25	0.091
Albumin (g/L)		37.48 ± 4.36	37.29 ± 5.30	37.29 ± 5.69	0.895
Globulin (g/L)		28.17 ± 5.11	28.02 ± 5.62	27.84 ± 4.64	0.837
Hemoglobin (g/L)		130.95 ± 17.68	130.69 ± 20.87	128.91 ± 20.56	0.633
Prealbumin (g/L)		138.87 ± 57.59	134.52 ± 57.08	138.04 ± 62.13	0.743
Fibrinogen (g/L)		2.84 ± 0.97	2.77 ± 0.84	2.91 ± 1.01	0.48
ALT (U/L)		30.07 ± 17.01	33.52 ± 23.01	32.62 ± 22.29	0.153
AST (U/L)		31.17 ± 15.26	31.92 ± 12.51	33.56 ± 17.39	0.362
AST/ALT		1.18 ± 0.52	1.43 ± 3.06	2.47 ± 12.47	0.1
GGT (U/L)		59.83 ± 48.94	64.16 ± 55.12	64.00 ± 60.70	0.618
ALP (U/L)		84.79 ± 32.34	85.15 ± 28.88	90.26 ± 37.54	0.314
TBIL (μmol/L)		18.38 ± 8.94	19.21 ± 9.49	19.90 ± 10.88	0.309
DBIL (μmol/L)		6.07 ± 4.01	6.68 ± 4.58	6.50 ± 4.50	0.293
Bile acid (μmol/L)		19.88 ± 28.41	23.41 ± 29.65	19.32 ± 23.69	0.376
Uric acid (μmol/L)		280.53 ± 89.76	269.55 ± 82.99	276.95 ± 89.79	0.441
Glucose (mmol/L)		5.81 ± 1.80	7.99 ± 24.35	7.66 ± 16.71	0.208
Cholesterol (mmol/L)		3.83 ± 0.91	3.92 ± 1.99	4.13 ± 2.44	0.244
Potassium (mmol/L)		3.95 ± 0.39	3.99 ± 0.38	3.98 ± 0.41	0.549
Sodium (mmol/L)		139.83 ± 2.65	138.95 ± 11.40	138.30 ± 13.71	0.205
Chloride (mmol/L)		103.80 ± 3.31	104.05 ± 4.46	104.24 ± 4.61	0.562
PT (s)		12.53 ± 1.50	12.83 ± 1.66	12.58 ± 1.32	0.127
APTT (s)		33.10 ± 4.54	33.59 ± 5.29	33.67 ± 4.55	0.407
TT (s)		15.66 ± 2.23	16.05 ± 2.10	15.86 ± 2.13	0.172
AFP (ng/mL)		225.24 ± 811.71	633.99 ± 3530.27	676.78 ± 2552.25	0.055

BCLC, Barcelona Clinic Liver Cancer; WBC, white blood cell; RBC, red blood cell; ALT, alanine aminotransferase; AST, aspartate aminotransferase; GGT, gamma glutamyl transpeptidase; ALP, alkaline phosphatase; TBIL, total bilirubin; DBIL, direct bilirubin; PT, prothrombin time; APTT, activated partial thromboplastin time; TT, thrombin time; AFP, alpha-fetoprotein.

### Identify risk factors affecting RFS through LASSO, RSF, and multivariate Cox regression analysis

**Figure 1:**
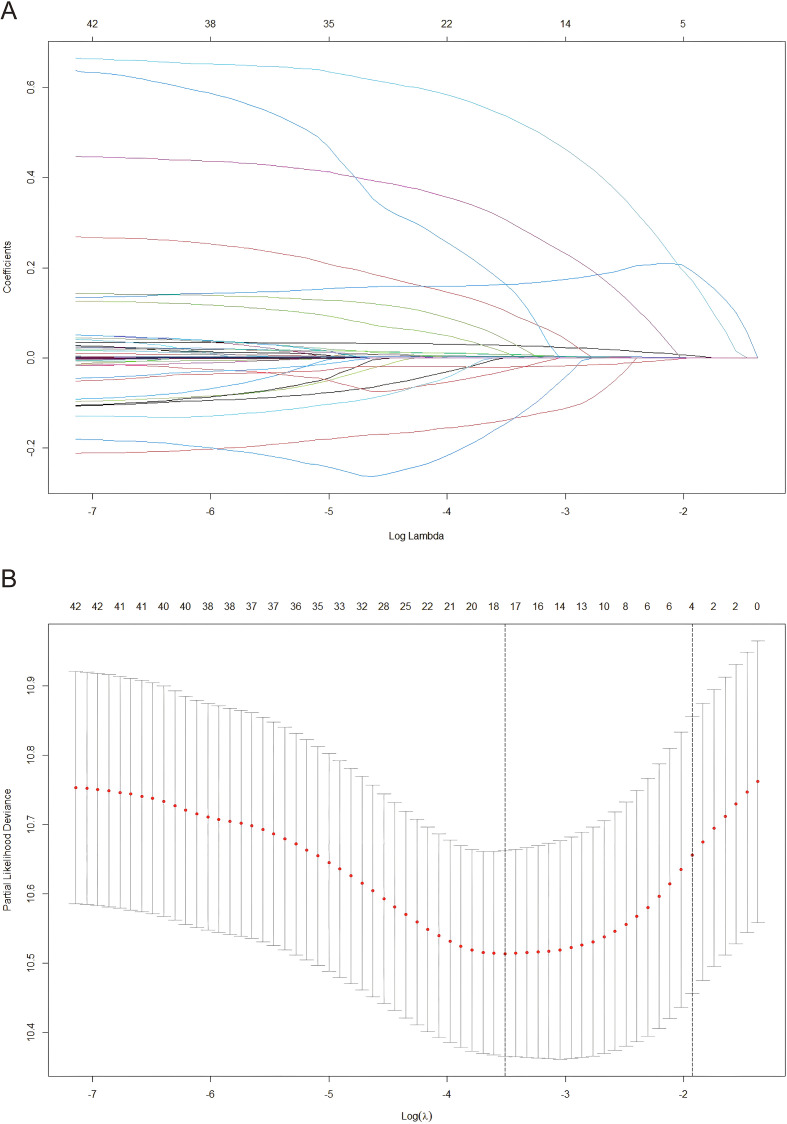
Variable screening for RFS via LASSO regression. RFS, recurrence-free survival; LASSO, least absolute shrinkage and selection operator.

To identify independent risk factors for RFS in HBV-related HCC patients after combination treatment, we applied LASSO regression, RSF, and multivariate Cox regression analysis. 42 clinical variables were initially included. LASSO regression with 10-fold cross-validation was first performed to reduce variable dimensionality and avoid overfitting. The optimal λ value (λ=0.030) was determined by the minimum partial likelihood deviance, retaining 17 variables: age, gender, hypertension, cirrhosis, BCLC, tumor number, tumor size, neutrophil, lymphocyte, monocyte, ALT, AST/ALT, TBIL, albumin, globulin, GGT, and APTT. These variables showed non-zero coefficients, indicating their potential prognostic value (**Figure 1**).

**Figure 2:**
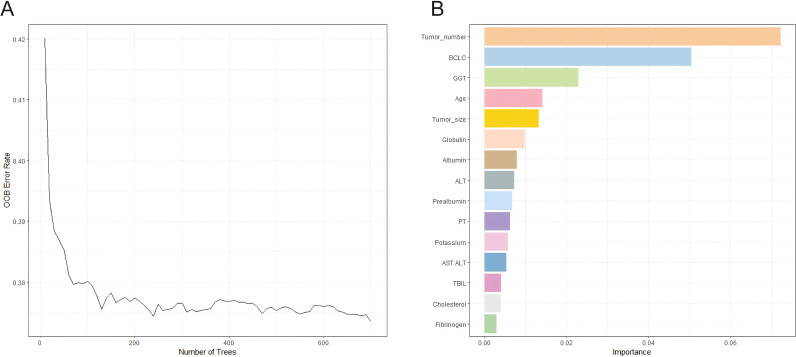
Variable screening for RFS via random survival forest. RFS, recurrence-free survival.

RSF was then employed to evaluate the prognostic importance of all 42 variables. This non-parametric approach effectively captures non-linear relationships and variable interactions without prior assumptions, providing a ranking of variable importance based on the minimal depth principle. The top 15 variables with the highest importance scores were selected for subsequent analysis, including tumor number, BCLC, GGT, age, tumor size, globulin, albumin, ALT, prealbumin, PT, potassium, AST/ALT, TBIL, cholesterol, and fibrinogen (**Figure 2**).

**Figure 3:**
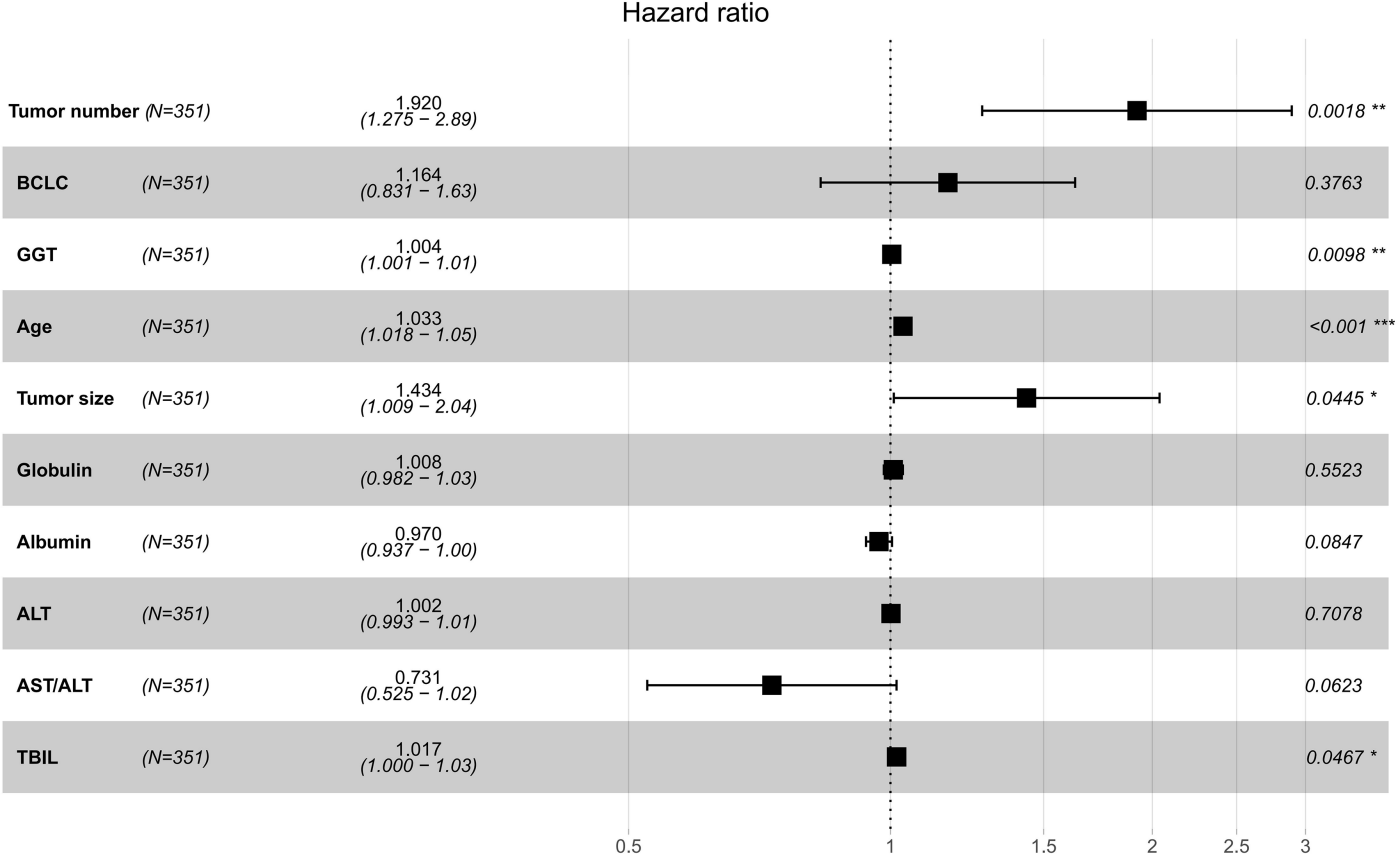
Variable screening for RFS via multivariate Cox regression analysis. RFS, recurrence-free survival.

The intersection of variables selected by the LASSO algorithm and those ranked within the top 15 by the RSF was then determined, consisting of tumor number, BCLC, GGT, age, tumor size, globulin, albumin, ALT, AST/ALT, and TBIL. This consensus approach, leveraging both penalized regression and ensemble tree-based methods, aimed to isolate a core set of features that were consistently deemed prognostically significant across different analytical frameworks, thereby enhancing the reliability of the findings. The variables common to both selection processes were subsequently entered into a multivariate Cox proportional hazards regression model for final confirmation and hazard ratio estimation. This final step allowed for the precise quantification of the independent association between each selected risk factor and RFS, while controlling for the simultaneous effects of all other factors in the consensus set, ultimately defining the foundational predictors for the subsequent construction of the prognostic nomogram. Ultimately, through multivariate Cox analysis, tumor number (P = 0.0018), GGT (P = 0.0098), age (P<0.001), tumor size (P = 0.0445), and TBIL (P = 0.0467) were identified as independent risk factors for RFS (**Figure 3**).

### Develop RFS nomogram based on the identified risk factors

**Figure 4:**
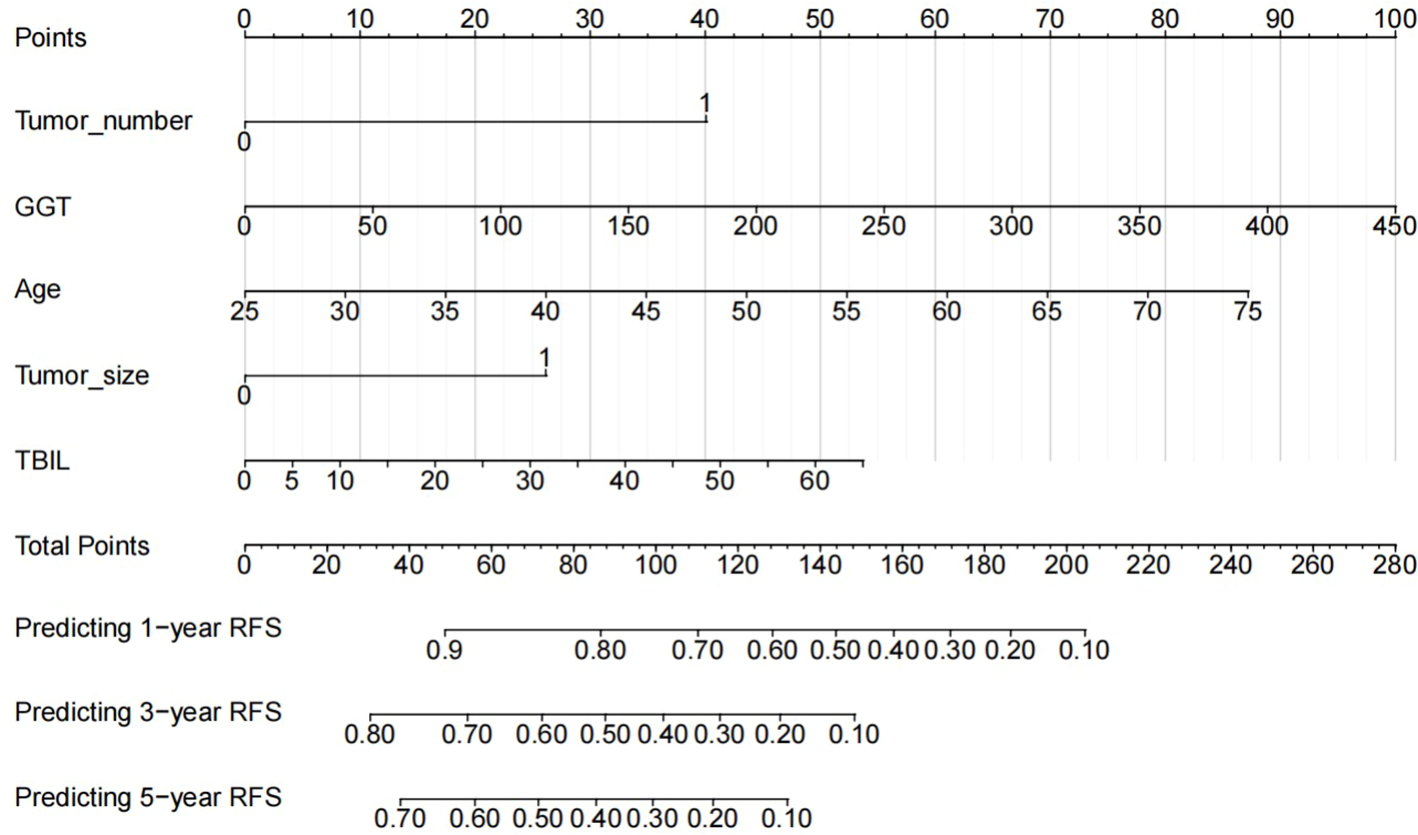
Nomogram developed for predicting 1-, 3-, and 5-year RFS of HBV-related HCC patients after TACE combined with RFA treatment. RFS, recurrence-free survival; HBV, hepatitis B virus; HCC, hepatocellular carcinoma; TACE, transarterial chemoembolization; RFA, radiofrequency ablation.

To visualize the prognostic model, a nomogram (**Figure 4**) was constructed incorporating the independent risk factors (tumor number, GGT, age, tumor size, TBIL). Each factor corresponds to a point value on the nomogram. Summing these yields a total points score, which directly maps to the predicted 1-year, 3-year, and 5-year RFS probabilities. This nomogram provides a user-friendly tool for individualized recurrence risk assessment.

**Figure 5:**
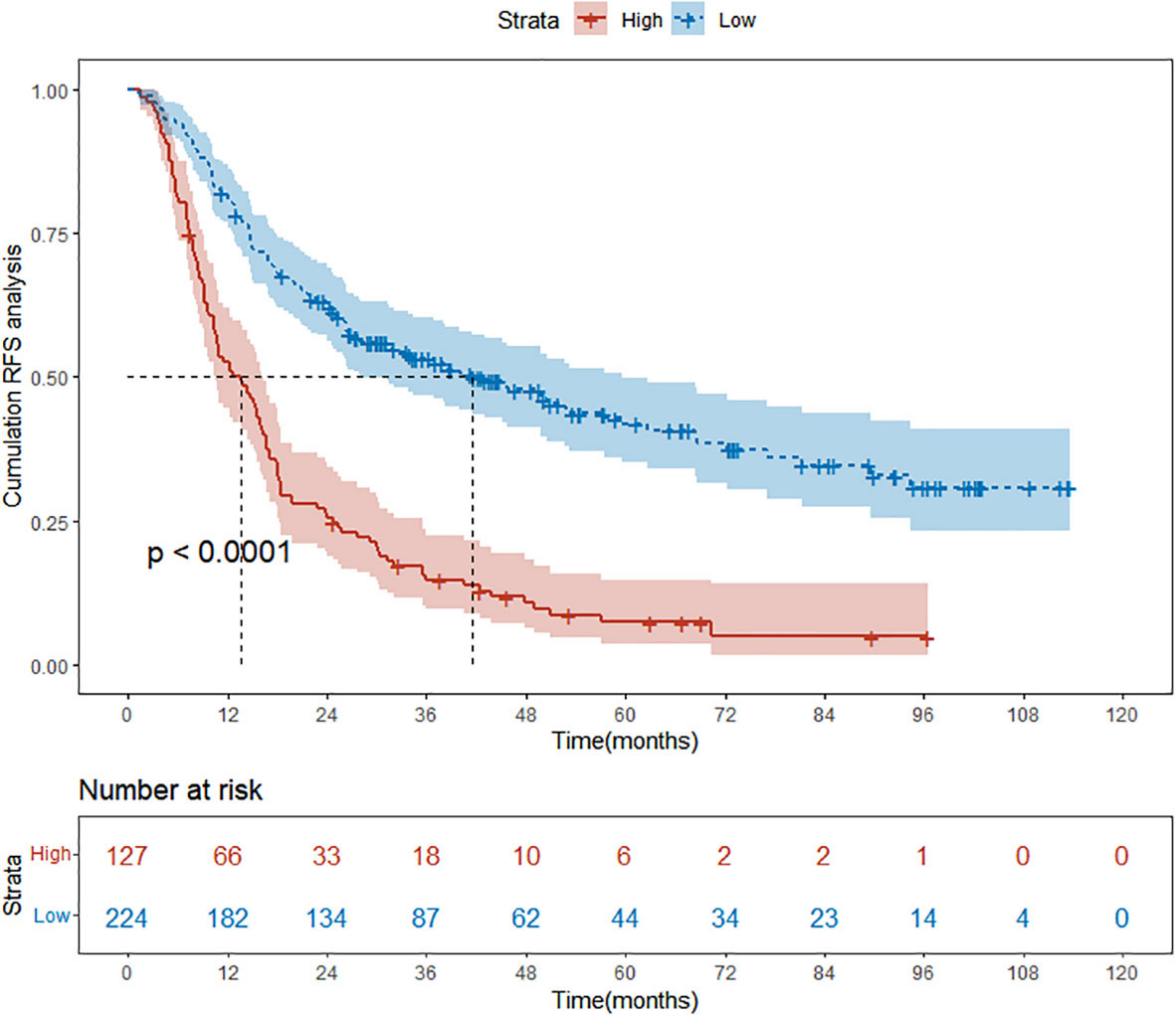
Kaplan-Meier curves of low and high risk groups stratified by nomogram-derived points in the training cohort.

**Figure 6:**
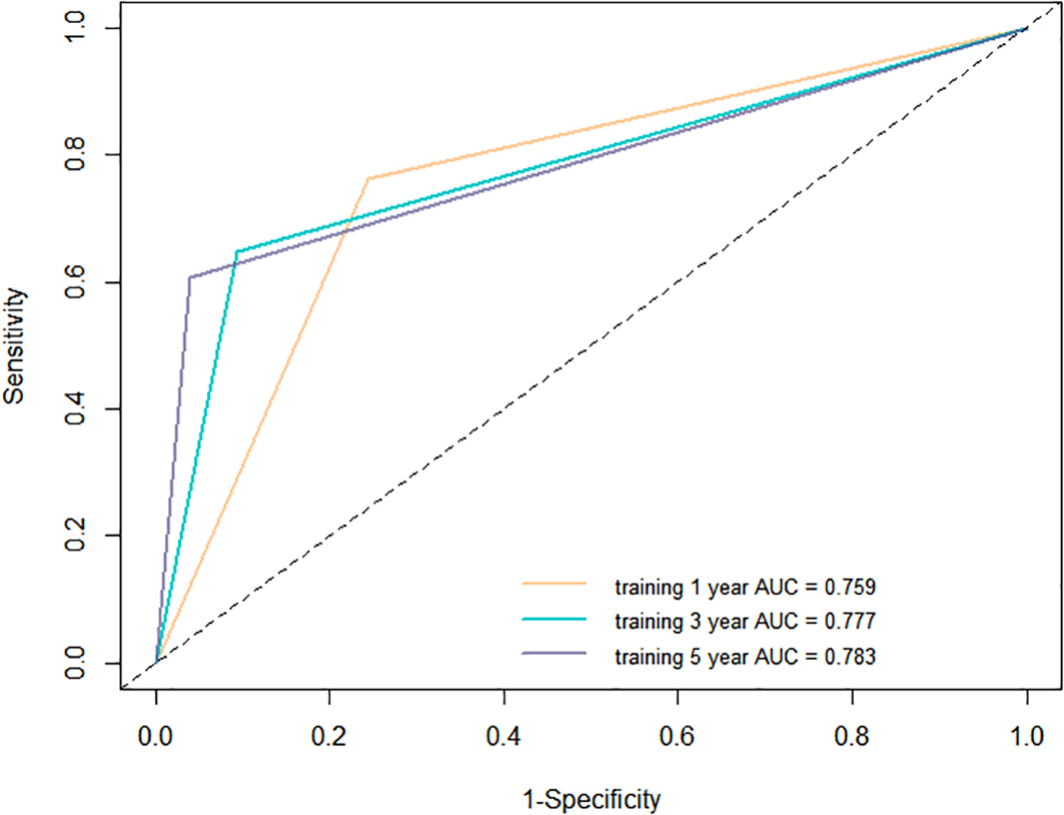
ROC curve analysis for 1-year, 3-year, and 5-year RFS prediction in the training cohort. ROC, receiver operating characteristic; RFS, recurrence-free survival.

We assessed the performance of the nomogram via Kaplan-Meier survival curves, ROC, and DCA analysis in the training cohort. First, patients were stratified into high- and low-risk groups using the optimal cut-off value of total points from the nomogram. Kaplan-Meier curves (**Figure 5**) revealed a significant divergence in RFS between the two groups (P < 0.0001), with the high-risk group (red curve) exhibited a drastically faster decline in cumulative RFS probability compared to the low-risk group (blue curve), confirming the nomogram’s capacity for effective prognostic stratification. Next, we evaluated the nomogram’s discriminative ability using ROC curves (**Figure 6**). The curves for 1-year, 3-year, and 5-year RFS (represented by orange, blue, and purple lines, respectively) all deviated substantially from the diagonal reference line (which denotes no predictive value). Corresponding area under the curve (AUC) values were 0.759 (1-year), 0.777 (3-year), and 0.783 (5-year), indicate good predictive accuracy.

**Figure 7:**
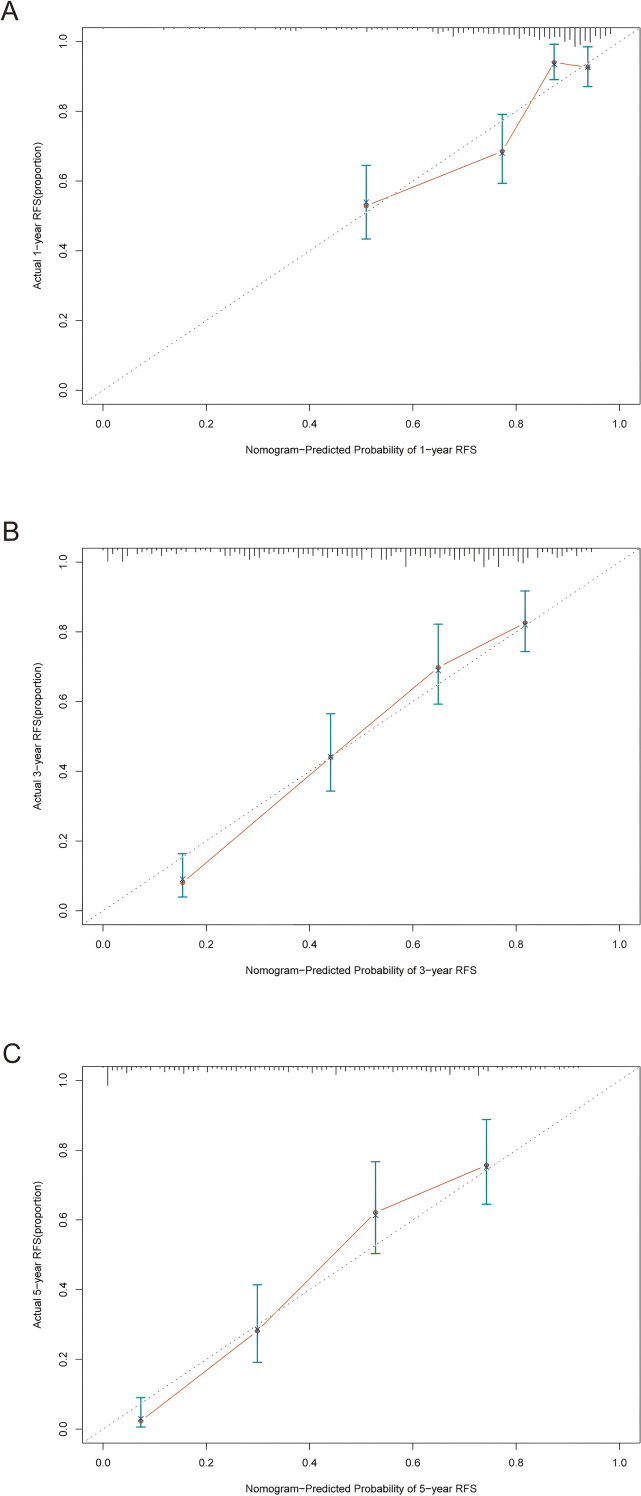
Calibration curves for 1-year **(A)**, 3-year **(B)**, and 5-year **(C)** RFS prediction in the training cohort. RFS, recurrence-free survival.

**Figure 8:**
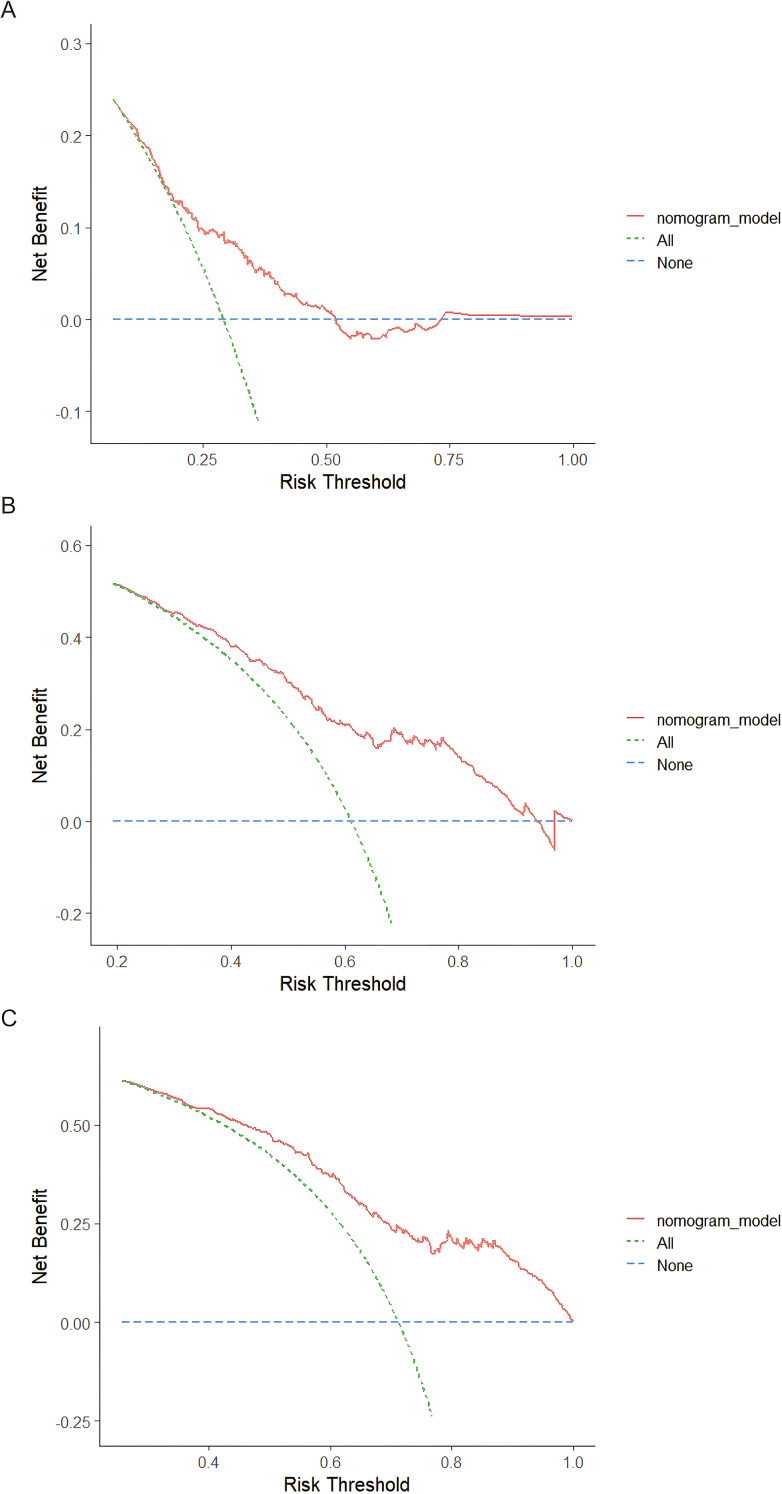
Decision curve analysis for 1-year **(A)**, 3-year **(B)**, and 5-year **(C)** RFS prediction in the training cohort. RFS, recurrence-free survival.

Then, the calibration curves for predicting 1-year, 3-year, and 5-year RFS demonstrated well agreement between the nomogram-predicted probabilities and the actual observations (**Figure 7**), suggesting that the nomogram possesses high accuracy and reliability in estimating outcomes. Finally, DCA was performed to quantify the clinical utility of the nomogram for 1-year, 3-year, and 5-year RFS (**Figure 8**). For all three time points, the nomogram model (red curve) consistently yielded positive net benefit across a broad range of risk thresholds, outperforming both the “treat all” (green dashed curve) and “treat none” (blue dashed curve) strategies, confirming that applying the nomogram to guide clinical decision-making provides meaningful outcomes.

### Validate RFS nomogram through the internal and external validation cohorts

We validated the nomogram performance in both internal and external cohorts using Kaplan-Meier survival curves and ROC analysis. In the internal validation cohort, the Kaplan-Meier curves (**Supplementary Figure S1**) showed a significant separation in RFS between the two risk groups (P < 0.0001). Consistent findings were observed in the external validation cohort (**Supplementary Figure S2**), where a statistically significant difference in RFS was also detected (P = 0.0008), confirming the nomogram’s robust prognostic stratification ability across distinct patient populations. Regarding ROC curve analysis for discriminative ability, time-dependent ROC curves were generated for 1-year, 3-year, and 5-year RFS in both cohorts. In the internal validation cohort, the AUC values were 0.708 (1-year), 0.751 (3-year), and 0.714 (5-year) (**Supplementary Figure S3**), while the external validation cohort yielded AUC values of 0.701 (1-year), 0.708 (3-year), and 0.751 (5-year) (**Supplementary Figure S4**). These results indicated sustained good to acceptable predictive accuracy of the nomogram in both internal and external settings.

## Discussion

In this multicenter study, we developed and validated a novel prognostic nomogram model for predicting RFS in HBV-related HCC patients after receiving TACE combined with RFA treatment. The model, incorporating five readily available pre-treatment variable - tumor number, GGT level, age, tumor size, and TBIL level - demonstrated consistent and significant predictive performance across both internal and external validation cohorts. Our findings underscore the intricate interplay between tumor burden, hepatic function, and host factors in determining the risk of recurrence.

Tumor-related parameters, including tumor number and tumor size, are well-established prognostic factors in HCC, and their inclusion in our model aligns with existing evidence (17–20). Multiple tumors and larger tumor sizes suggest higher recurrence rates and poorer overall survival. GGT and TBIL, two liver function and biliary tract markers, adds a unique dimension to our prognostic model by linking hepatic reserve to recurrence risk. GGT is an enzyme involved in glutathione metabolism, and its elevation in HCC patients has been associated with oxidative stress, epithelial-mesenchymal transition, and invasiveness of tumor cells (21–24). In liver disease, GGT elevation also reflects chronic hepatic inflammation, which create a microenvironment conducive to tumor progression and recurrence (25). Our finding that GGT is an independent predictor of RFS is consistent with some meta-analysis, which reported that elevated GGT levels were associated with poor prognosis in HCC patients (26, 27). TBIL, a classic marker of hepatocyte dysfunction and cholestasis, is also a well-documented prognostic factor in HCC (28, 29). Elevated bilirubin level indicates impaired liver synthetic function, which may increase the risk of treatment-related complications (30). Age is also closely associated with cancer prognosis. However, the exclusion of alpha-fetoprotein (AFP) as an independent predictor in our final model is noteworthy. While AFP is a classic biomarker, its prognostic value may have been overshadowed by more direct markers of liver injury and tumor volume in this specific population receiving combined therapy. Similarly, although some inflammatory markers and blood cell counts were initially screened, they did not retain significance in the multivariate analysis.

The liver is a specialized immunological organ where hepatocytes, sinusoidal endothelial cells, and Kupffer cells constantly process and present antigens via major histocompatibility complex (MHC) class I and II pathways (31). In HBV-related HCC, this system is chronically dysregulated. Persistent HBV infection leads to aberrant viral antigen presentation, contributing to T cell exhaustion and immune escape (32). While distinct from autoimmune hepatitis (AIH), where MHC-mediated presentation of autoantigens triggers autoreactive lymphocyte activation and progressive injury (33), HBV-related HCC shares a common theme of immune-mediated remodeling. Sustained inflammation may alter the hepatic microenvironment, creating a “pre-malignant niche” conducive to recurrence. While this study does not directly assess these parameters, it provides directions for future research integrating them, especially as immunotherapy becomes increasingly important in HCC.

Our prognostic model has several major advantages. Unlike single-center studies that are limited in reflecting the heterogeneity of clinical practice, a key strength of our study lies in its multicenter design, which substantially improves the external validity and generalizability of the proposed model. Secondly, in contrast to complex genomic or proteomic signatures that are costly and difficult to implement in routine clinical practice, our model is based on variables that are routinely measured during preoperative evaluation, making it highly accessible for widespread clinical application, particularly in resource-limited settings where advanced diagnostic technologies are not universally available. Numerous prognostic models for HCC exist, ranging from generic staging systems to models specific to surgical resection, ablation, or transplantation. The strength of our model also lies in its specificity and practicality. It is specifically tailored for HBV-related HCC patients receiving the combination treatment of TACE and RFA, a growing yet heterogeneous cohort that may not be perfectly adjudicated by generic systems.

Despite these strengths, our study has several limitations that warrant acknowledgment. While our multicenter approach enhances generalizability, retrospective cohorts are inherently susceptible to selection bias and unmeasured confounding. Notably, in the context of HBV-related HCC, long-term adherence to antiviral therapy and the subsequent virological response (e.g., HBV DNA suppression) are critical determinants of recurrence dynamics. The lack of granular data on long-term viral load fluctuations represents a limitation. These factors could potentially modulate the hepatic microenvironment and influence the frequency of recurrence. Secondly, the study is restricted to Chinese patients, and thus the model requires validation in other ethnic populations to confirm its global generalizability, and the inclusion of additional patients in future research could further strengthen the model’s statistical robustness. Thirdly, the long inclusion period of this study introduces the possibility of temporal bias. Over the past decade, advancements in imaging resolution, refinements in TACE and RFA techniques, and the introduction of more potent antiviral agents may have subtly shifted the baseline prognosis and treatment efficacy. While our model focuses on robust, routinely available pre-treatment variables to ensure clinical utility, future prospective studies are needed to evaluate how evolving treatment standards and modern imaging technologies might refine the predictive accuracy of these parameters across different eras of care. Furthermore, it should be emphasized that the nomogram constructed in this study is better considered a preliminary or intermediate prediction model, and further rigorous validation and comparative analysis with other clinical approaches that may arise in the future are still needed before it can be recommended for routine clinical application.

## Conclusion

This study successfully developed a well-performed nomogram model to predict 1-, 3-, and 5-year RFS in HBV-related HCC patients following TACE combined with RFA.

## Data Availability

The original contributions presented in the study are included in the article/**Supplementary Material**. Further inquiries can be directed to the corresponding authors.

## Supplementary material

Supplementary Figure S1 Kaplan-Meier curves of low and high risk groups stratified by nomogram-derived points in the internal validation cohort.

Supplementary Figure S2 Kaplan-Meier curves of low and high risk groups stratified by nomogram-derived points in the external validation cohort.

Supplementary Figure S3 ROC curve analysis for 1-year, 3-year, and 5-year RFS prediction in the internal validation cohort. ROC, receiver operating characteristic; RFS, recurrence-free survival.

Supplementary Figure S4 ROC curve analysis for 1-year, 3-year, and 5-year RFS prediction in the external validation cohort. ROC, receiver operating characteristic; RFS, recurrence-free survival.
