# Supplementary figures and images for: Prognostic model for predicting recurrence-free survival in HBV-related hepatocellular carcinoma patients after combined treatment: a multicenter study
Source: Front Oncol. 2026 May 4;16:1760126. doi: 10.3389/fonc.2026.1760126 (PMC13180601; doi:10.3389/fonc.2026.1760126)

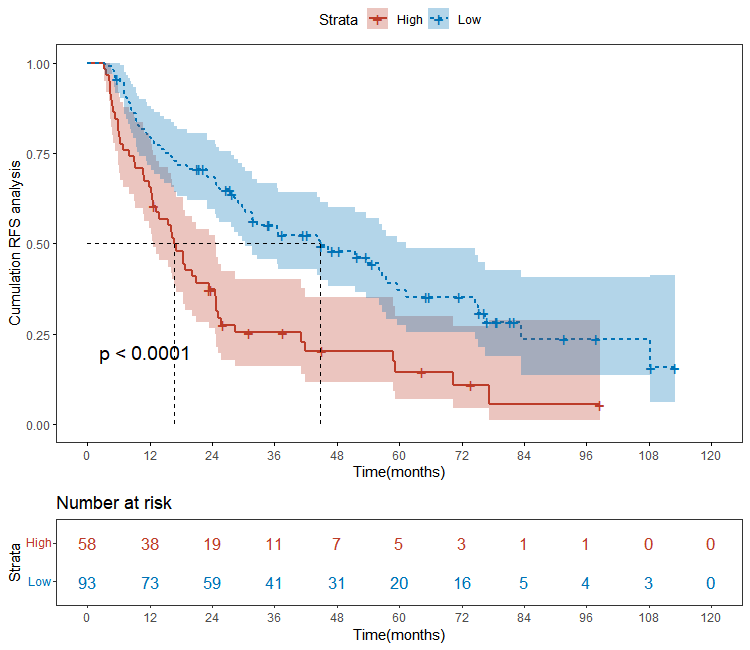

Supplement: Supplementary Figure S1 — Kaplan-Meier curves of low and high risk groups stratified by nomogram-derived points in the internal validation cohort. [file Image1.tiff]

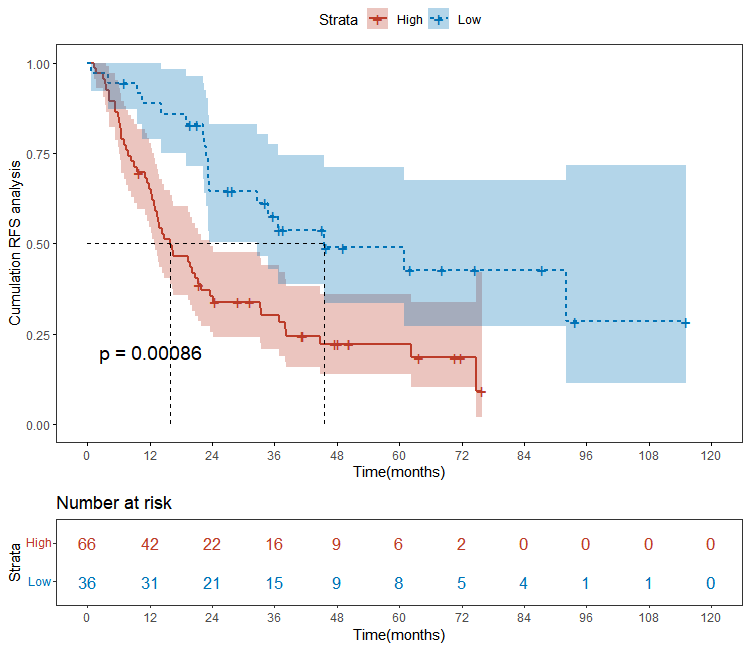

Supplement: Supplementary Figure S2 — Kaplan-Meier curves of low and high risk groups stratified by nomogram-derived points in the external validation cohort. [file Image2.tiff]

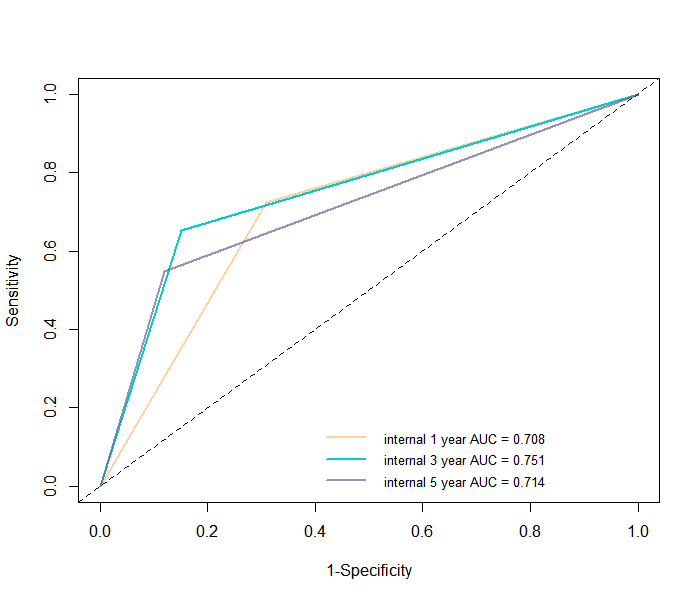

Supplement: Supplementary Figure S3 — ROC curve analysis for 1-year, 3-year, and 5-year RFS prediction in the internal validation cohort. ROC, receiver operating characteristic; RFS, recurrence-free survival. [file Image3.tiff]

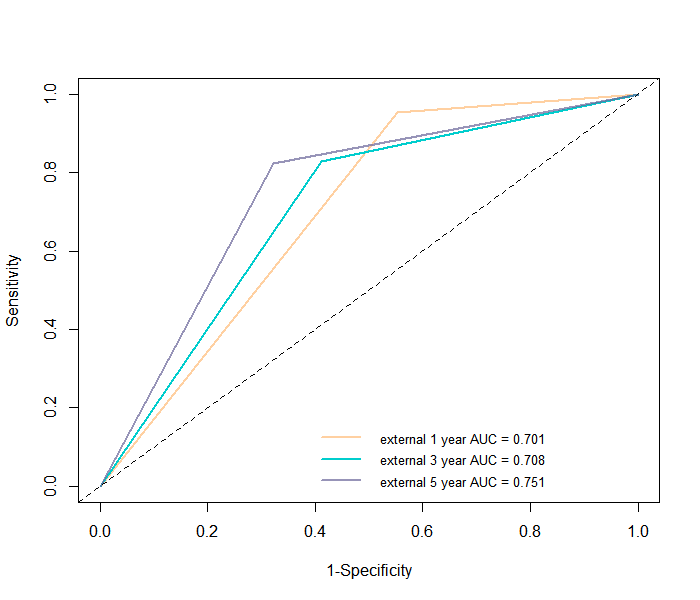

Supplement: Supplementary Figure S4 — ROC curve analysis for 1-year, 3-year, and 5-year RFS prediction in the external validation cohort. ROC, receiver operating characteristic; RFS, recurrence-free survival. [file Image4.tiff]
